# Students Address

**Published:** 1869-04

**Authors:** George F. Keesee

**Affiliations:** the Graduating Class.


### ARTICLE II.

### *Students Address.*

By George F. Keesee, D.D.S., of the Graduating Class.

Delivered at the Annual Commencement of the Baltimore College of Dental Surgery, March 3d, 1869.

*Honored Professors, Fellow Students, Ladies and Gentlemen:*

Your presence this evening, demands no ordinary tribute. The occasion discloses your ready homage to Science and Progress; and at the same time exhibits the amost divine sentiment in the human breast which prompts this hearty "God speed" to those who after this hour are to enter upon the sterner and more active duties and responsibilities of life. We most cordially reciprocate the sentiments which your presence embodies, and give you heartygreetings to this final scene of our student life.

The fact that the Dentist must live by the *bad things* which he extracts from other people's mouths, does not necessarily impose on him the duty of disgorging *good things* when occasion requires that his mouth should be opened. The recency of our induction into titled honors of the science of Dental Surgery, will be a sufficient apology, on the part of a mere novitiate, for dwelling a few moments upon *Teeth and Dentists.*

Science no longer demands defenders; her worshippers are not the consorts of demons. The student of nature, even in popular estimation, is no longer in league with the Prince of Darkness. The powers, and secret forces of *Nature*, become the powers of *Man* in the work of progress; and as man advances, the purposes of God are manifested. His goodness vindicated, and His wisdom exalted.

The very spirit of science and art is progressive. Nature has storehouses yet unexplored, and the ready intelligence of man discovers facts that his more laborious study must render available, practical and systematic.

In *no* field of investigation and experiment has the intelligence and toil of man been better rewarded than in the study of Medicine and Surgery in all their various branches and applications. For centuries the progress of these departments of learning was slow, uncertain, and unsatisfactory. The *Materia Medica* was limited to a few herbs and plants, and to these superstition lent a charm which made them efficient. Latterly the progress of these studies has kept pace with the grand advance of other sciences. So long as man believed that the forces of nature would apply themselves to the most beneficient ends in human economy, without the impulse of the human mind there was no advance; for the very thought that matter could accomplish this, implied the miserable fallacy that matter and mind are one. While we may grant that *mind* actually *creates* nothing; and that the poet only deals with material which memory has treasured up, yet the *form* imparted by the poet is new; and the whole universe, so far as known to us, shows no example where matter spontaneously springs into those endlessly diversified shapes and forms which it assumes under the plastic hand of man, guided by the power of mind. The dumb, blind and silent elements of nature have been vanquished in their own secret domain and become the vassals of man; the slaves of the invisible power—*mind.* Nature does not reply until interrogated, nor yield till her secrets are extorted. As soon might we hope to see the very soil and rocks beneath our feet spring, by magic, into this beautiful city, as expect to see the dormant forces of nature reveal themselves without the power of mind. There is no greater triumph of human intellect than is manifest in the subordination of the forces of nature to man's purposes in acquiring an almost complete victory over *human pain.* A triumph which makes the sufferer laugh at his own sufferings: even more than that; to be unconscious of his own mutilation and torture. The sublime achievements of human genius by which the lightning is made a slave and obedient to man's behests, nor the scarcely less sublime victory by which he sends stately ships across the waves, defying tide and tempest, is not a greater triumph of intelligence than the victory acquired over bodily pain. Experience teaches this singular lesson, that the *degree of sensibility* to pain is increasing in direct proportion with the increase of civilization; and to an extent, perhaps, that mere casual observers have not noted.

The connection between mind and body is so intimate that the point of union cannot be distinctly marked by us; we cannot say that mind ends *here*, and body commences *there*, yet the connection is universally acknowledged, and by common consent the healthy condition of the one is a necessary condition of the other. More especially is it conceded as true that a sound mind requires a sound body. It is not wonderful then that, as man progresses in intellectual development he becomes more keenly sensitive to esthetical beauties, or blemishes, and as a sort of consequence that his physical nature becomes more keenly sensitive to bodily pleasure and pain. *Hence* the necessity of meeting the advance of suffering by corresponding advances in the healing art.

Observation discloses the fact that the very signs and expressions by which pain is manifested are deceptive. Chloroform not only relieves the patient of bodily pain, but of all sensation, and even while the sufferer from epilepsy exhibits most violent writhings and contortions in his fearful and appalling spasms, it is known that the very initial step in the train of phenomena which prostrates him, is an *absolute loss* of consciousness, and of course all the subsequent exhibitions of suffering are merely bodily extortions without a particle of conscious sensibility to suffering or pain.

The domain of science is too broad, and even a single branch is too extensive to allow any *one person* to attain a complete mastery of all its parts and details in the course of an ordinary life-time. The *thoroughness*, which is as characteristic of the age as is progress itself, imperatively demands division of labor, and the apportionment of special departments ot Medicine and Surgery to different individuals. In this distribution, surgery itself may be subdivided into distinct departments. This demand is now being met in the very rapid growth of a class of men known as *Dental Surgeons.* The most popular idea of a dentist is that he is a necessary evil, and that society tolerates *him* because neither society nor philosophers can tolerate the *tooth-ache.* His operating chair, in vulgar estimation, is a refinement of the guillotine; his forceps the very quintessence of a inquisitorial armory, and his dainty files and drills and excavators just so many cunningly devised instruments for inflicting the most exquisite torture on sensitive and irritated nerves. The dentist himself is but a merciless inflictor of the keenest pain. What cares he for the twitching of muscles, the snapping of nerves or the shrieks and cries of the victim in his chair? He can smile when he convulses you with agony, and declare that you *do not* suffer when every fibre in your body is shivering with pain. In the pride of his delicate manipulations he defiantly avers that pain is impossible. Dentists would never convert the world into Berkleyites. This opinion is founded on the presumption that pain admits of no alleviation from *art*; as if sorrow found no relief in the delicate refinements of sympathy; as though " Our sweetest song could not tell of saddest thought."

Poetry has done much for the color of eyes, black and blue. Noses perhaps have not had their due mead of praise; but it is certain that others besides poets have gone mad over the sparkling pearls within the lips they love, and have indulged in transports that *praise*, and *verse* and *song* have never reached. The dentist is no foe of beauty because he would aid the work of nature. Not for the love of science would he rob loveliness of a charm; nor poesy of a theme. An ancient and well authorized classification of teeth, *grammatically* speaking is into *Regular*, *Irregular* and *Defective*, The business of the dental profession is to *preserve* the regular, to *arrange* and *beautify* the irregular, and to *remove* and *substitute* the defective. He would not mar a line or tint of beauty where it exists; he would rather remove the deformity that renders the laugh hideous, and supply the snowy pearl that lends enchantment to the bewitching smile. He would give rotundity to the sunken cheek, restore the lines of beauty to the compressed lips, impart the fragrance of spring's sweetest flowers to the breath, and invest matrons and maidens, the old bachelor and the buxum beau with the fascination and charm which always attach themselves to the regular rows of shining pearls within the ruby lips.

As *humanitarians* we would relieve pain; as *artists* we would remove deformity and beautify the human face divine; and as *professional* men we would seek a just return for the outlay in preparing for this work, and for the skill with which we perform it.

To elevate the profession, and instil a love of the correct principles of science has been the aim of our instructors; and the reflection must be most gratifying that the fame of the institution over which they preside has become more than *national*. The young men of other civilized nations now seek their presence to enjoy the advantages which this college presents.

Our country has reason to be proud that her sons meet on equal ground the leaders in science and of the mechanical arts of other countries. Throughout the civilized world *American* genius and skill and research are regarded as important factors in working out the great problems of the world and age.

In severing our connection with this institution, and in leaving you, honored instructors and friends, we feel that you have a legacy in the well-won reputation accorded by an enlightened world, which must compensate you for the labors you have bestowed upon this class, and the inefficiency of our love and gratitude to discharge the debt.

The young men who enjoy the instruction of such Professors in their several departments as PIGGOT in Chemistry, of NOEL in Physiology, of HOWARD in Anatomy, of GORGAS in Dental Surgery, of AUSTEN in Mechanical Dentistry, and BOND (last, not least,) in Pathology and Therapeutics, must either be deficient in ability, or criminally remiss in application and study, or they must go forth from the Halls of the Dental College of Baltimore thoroughly qualified for the work of the Dental profession. We shall cherish their names on our Diplomas; recall with pleasure our social intercourse with them, and strive to fill our profession in such a manner as will reflect credit on *them*, and upon the honored institution which they represent.

May the *Bond* that binds us together never slacken with age, and may the burnished images of PIGGOT, GORGAS, AUSTEN, HOWARD, NOEL, GRANT and WATERS remain untarnished upcn the beautiful clasp.

I feel that I would betray the trust committed to me were I not in these few passing remarks to pay a tribute of respect to one whom we had learned to honor and reverence. I refer to Professor A. SNOWDEN PIGGOT. For several months past, we as students had been brought into social and professional intercourse with Professsor Piggot and had always found in him one affable in his manners, and ready and willing at all times to impart the instructions for which we sought his presence. The Baltimore Dental College by the death of so distinguished and influential a member of its Faculty has sustained a loss from which it cannot speedily recover. To fill the Chair of Chemistry requires, under the most favorable circumstances, a man of untiring zeal and energy, more especially is this the case at the present, when the chemical and analytical world is undergoing so perfect a revolution. But in the person of him whose death we deplore there was found one equal to the task, for having by dilligent application of a coined brain become the equal and perhaps the surpasser of a large portion of his compeers, he sought for facts, and by practical demonstrations brought to the professional world a fund of knowledge richer by far than pearls from the mighty deep. But these whom I have mentioned have not alone been the sufferers, for an enlightened public having accorded to him distinguished honors in placing him also in a high position, now too join with us in deploring his untimely death. "Peace to his ashes."

To you fellow-students I have but a few words to say in parting, our competition has been honorable and we part as *friends*, feeling a mutual and reciprocal interest in each others future fortunes and success. May happiness and prosperity attend you in the pursuit of your profession, and may the sunset of life foretoken the oncoming of a blessed immortality in the better land. *Good-bye and God bless you.*

And to you generous citizens of a beautiful and prosperous city whose very name has become the synonym of hospitality and of all the social virtues, what shall I say? Words are powerless to express all we feel. *Baltimore* has laid the *whole South* under a debt of gratitude that it can never repay. There was a time when many of the *sons of the South* were hungry and you fed them; naked and you clothed them; strangers and you took them in; sick and imprisoned and you visited them. May Heaven reward you with happy homes; and may the blessing of a kind Providence attend your enterprise, success your commerce, and add new and increasing lustre to your fair city till the sunset of time.

---

